# Proof-of-Concept: Smartphone- and Cloud-Based Artificial Intelligence Quantitative Analysis System (SCAISY) for SARS-CoV-2-Specific IgG Antibody Lateral Flow Assays

**DOI:** 10.3390/bios13060623

**Published:** 2023-06-05

**Authors:** Samir Kumar, Taewoo Ko, Yeonghun Chae, Yuyeon Jang, Inha Lee, Ahyeon Lee, Sanghoon Shin, Myung-Hyun Nam, Byung Soo Kim, Hyun Sik Jun, Sungkyu Seo

**Affiliations:** 1 Department of Electronics and Information Engineering, Korea University, Sejong 30019, Republic of Korea; skumar@korea.ac.kr (S.K.); rdks95@korea.ac.kr (T.K.); ahyun904@korea.ac.kr (A.L.); ghost10s@korea.ac.kr (S.S.); 2 Season Co., Ltd., Sejong 30127, Republic of Korea; proin@season.co.kr; 3 Department of Biotechnology and Bioinformatics, Korea University, Sejong 30019, Republic of Korea; juy0811@korea.ac.kr (Y.J.); dlsgk1017@korea.ac.kr (I.L.); 4 Department of Laboratory Medicine, Anam Hospital, Korea University College of Medicine, Seoul 02841, Republic of Korea; yuret@korea.ac.kr; 5 Department of Hematology, Anam Hospital, Korea University College of Medicine, Seoul 02841, Republic of Korea; kbs0309@korea.ac.kr

**Keywords:** smartphone, point-of-care testing, lateral flow assay, artificial intelligence, SARS-CoV-2, IgG antibody

## Abstract

Smartphone-based point-of-care testing (POCT) is rapidly emerging as an alternative to traditional screening and laboratory testing, particularly in resource-limited settings. In this proof-of-concept study, we present a smartphone- and cloud-based artificial intelligence quantitative analysis system (SCAISY) for relative quantification of SARS-CoV-2-specific IgG antibody lateral flow assays that enables rapid evaluation (<60 s) of test strips. By capturing an image with a smartphone camera, SCAISY quantitatively analyzes antibody levels and provides results to the user. We analyzed changes in antibody levels over time in more than 248 individuals, including vaccine type, number of doses, and infection status, with a standard deviation of less than 10%. We also tracked antibody levels in six participants before and after SARS-CoV-2 infection. Finally, we examined the effects of lighting conditions, camera angle, and smartphone type to ensure consistency and reproducibility. We found that images acquired between 45° and 90° provided accurate results with a small standard deviation and that all illumination conditions provided essentially identical results within the standard deviation. A statistically significant correlation was observed (Spearman correlation coefficient: 0.59, *p* = 0.008; Pearson correlation coefficient: 0.56, *p* = 0.012) between the OD450 values of the enzyme-linked immunosorbent assay and the antibody levels obtained by SCAISY. This study suggests that SCAISY is a simple and powerful tool for real-time public health surveillance, enabling the acceleration of quantifying SARS-CoV-2-specific antibodies generated by either vaccination or infection and tracking of personal immunity levels.

## 1. Introduction

Since December 2019, the deadly severe acute respiratory syndrome coronavirus 2 (SARS-CoV-2) has infected more than 762 million people, led to approximately seven million deaths, and caused unprecedented disruptions to public health and the economy [1,2,3]. Real-time polymerase chain reaction (RT-PCR) for the detection of viral nucleic acids is currently the gold standard for the diagnosis of coronavirus disease 2019 (COVID-19) [4,5]. However, practical limitations prevent it from keeping pace with the rapid spread of the virus. For example, collecting samples from the nasopharynx is a painful process that can take a long time and requires licensed laboratories and specialized skills [6]. Therefore, RT-PCR is not suitable for rapid and straightforward patient screening. Rapid screening to identify SARS-CoV-2-infected patients is essential to prevent transmission of the virus, ensure timely treatment, and support mass testing. Considering these factors, the focus has shifted to point-of-care testing (POCT) because it is easy and quick to perform and can screen large numbers of patients [7,8,9,10,11,12,13]. In addition, experts agree that mortality rates can be reduced if more people are vaccinated, and that complete elimination of COVID-19 may not be possible until all people have access to vaccination. Despite the availability of 13.33 billion vaccine doses worldwide over the past two years, by April 2023, less than half of World Health Organization member countries (WHO) have reached the organization’s goal of 70%, and only 37% of health professionals in low-income countries have received primary vaccination [14,15]. Booster vaccinations and additional doses account for approximately 20% of all administered vaccinations today [16]. According to the director general of the WHO, Tedros Adhanom Ghebreyesus, “Blanket booster programs are likely to prolong the pandemic, rather than ending it, by diverting supply to countries that already have high levels of vaccination coverage, giving the virus more opportunity to spread and mutate” [17]. In addition, there is the question of vaccination parity—who should receive booster vaccinations and when. 

Various techniques have been developed for the detection of SARS-CoV-2, including nucleic acid amplification tests (NAATs), antigen tests, and antibody tests [18,19]. NAATs, such as polymerase chain reaction (PCR) and loop-mediated isothermal amplification (LAMP), are the most commonly used techniques for detecting SARS-CoV-2 [20]. These techniques amplify viral RNA to a detectable level and can provide highly sensitive and specific detection of the virus. PCR and LAMP have been shown to have comparable sensitivity of 95–100% [21,22,23]. However, their high cost and complexity make them unsuitable for rapid POCT. Antigen testing is another technique used to detect SARS-CoV-2. In this technique, a sample is tested for the presence of viral proteins using specific antibodies. Antigen tests are rapid and can provide results within 15 to 30 min. However, the sensitivity (50–90%) of antigen tests is lower than that of NAATs [24]. Antibody tests are techniques that detect the presence of SARS-CoV-2-specific antibodies in the human body. These tests can be either rapid diagnostic tests (RDTs) or laboratory-based tests. RDTs are lateral flow assays that provide results within minutes, while laboratory-based tests use enzyme-linked immunosorbent assay (ELISA) or chemiluminescent immunoassay (CLIA) techniques and require specialized equipment. The sensitivity and specificity of antibody tests vary depending on the type of antibody tested and the timing of the test. The sensitivity of antibody tests for the detection of SARS-CoV-2 has been reported to range from 30.1% for IgM tests to 72.2% for IgG tests when samples are collected within the first week of symptom onset. The sensitivity of antibody tests increases over time, with IgM tests having a sensitivity of 73.9% and IgG tests having a sensitivity of 91.4% when samples were collected 15–35 days after the onset of symptoms [25].

During the COVID-19 pandemic, there has been an urgent need for rapid and accurate testing methods to detect SARS-CoV-2 infection. Immunodiagnostic kits that detect IgM/IgG antibodies formed by vaccination or infection have been developed to qualitatively identify the presence of antibodies in patient samples [13,26,27]. Although the concentrations of IgG and IgM antibodies can provide information about a person’s immune response to infection, they do not necessarily reflect the person’s immunity to the pathogen or ability to prevent or control infection. Nevertheless, detection of IgG and IgM antibodies to SARS-CoV-2 has been shown to be a reliable indicator of infection. IgM antibodies can be detected in the blood of patients at 3–6 days and IgG at 8 days after COVID-19 infection [10]. Detection of both IgM and IgG antibodies could provide valuable information about the time course of viral infection, and rapid detection of these antibodies could improve the diagnosis and treatment of COVID-19.

One of the promising methods for the POC detection of IgM/IgG antibodies in patient samples is the lateral flow assay (LFA), which is based on the light scattering phenomenon of nanoparticles. In this method, antibodies bind to gold nanoparticles (GNP) conjugated to a non-pathogenic antigen and move in a specific direction [28,29]. LFA tests are commonly used at the POC because they are user-friendly, allow on-site testing, and provide results within minutes, making them cost-effective [30,31]. However, rapid LFA tests can only provide qualitative information about the presence or absence of antibodies, rather than quantification of specific antibodies.

The conventional methods of measuring an LFA signal use clinical or research laboratory equipment, such as a spectrophotometer or a fluorometer, which provide exceptional performance. These instruments provide accurate and precise measurements of signal intensity and can be used to quantify the amount of analyte present in the sample. However, the use of laboratory equipment can be expensive, bulky, time-consuming, and requires specialized training, making it difficult to use LFA assays in resource-limited settings or POC applications [32,33,34,35,36]. To overcome this limitation, several portable and handheld devices for LFA signal measurement have been developed. Some examples of portable devices for measuring LFA signals are the portable fluorescence readers, such as the Q-POC [37,38,39,40]. Additionally, smartphone-based systems are also being developed to provide a rapid, user-friendly, and cost-effective method for antibody quantification [41,42].

Several researchers have developed smartphone platforms to quantify various analytes, including concentrations of vitamin D, iron, and vitamin B12. Lee et al. and Srinivasan et al. developed a smartphone platform to quantify vitamin D and iron levels [43,44,45]. However, these systems usually require an external device connected to the smartphone, which limits the functionality and increases the cost. For example, Lee et al. developed a low-cost POC quantification system for vitamin B12 concentrations using silver amplification to increase the initial signal and detection limit within the required range [41]. However, they used a specially designed LFA kit with a “spacer pad” to extend the duration of the critical competitive binding reaction. Similarly, Foysal et al. developed a smartphone-based analyte detection method to measure albumin in human serum on an LFA strip [40]. However, this method cannot provide accurate data for slight misalignment under the camera. The LFA kits must be accurately placed in the field of view to avoid errors in the calculation of the number of pixels. Tong et al. developed an AI-assisted colorimetric LFA platform based on polydopamine nanoparticles using the receptor-binding domain of viral spike protein and mouse IgG for the quantification of neutralizing antibodies from vaccines [13]. They used a portable smartphone-based reader to image the LFIA results and output them to the AI algorithm to analyze the concentration of neutralizing antibodies. This approach is a promising solution for accurate quantification of LFAs using smartphones.

Here, we present a smartphone- and cloud-based artificial intelligence analysis system (SCAISY) for SARS-CoV-2-specific IgG antibodies detected on LFA strips. SCAISY allows users to measure and quantify antigen and SARS-CoV-2 antibody retention rates for various purposes based on the rapid LFA-type immunodiagnostic kit quickly and easily. This system provides statistical data in addition to antibody retention values without the need for an external device. In addition, SCAISY ensures robust performance under a wide range of illumination conditions and camera orientations without the need for an external illumination device. As demand for vaccination and immunity testing rapidly increases, SCAISY is a simple and powerful method to track vaccination effectiveness in large populations, especially in resource-limited countries. This technique can be used as a real-time public health tool to monitor immunity levels and determine the need for additional vaccinations and immunity to specific antigens based on vaccination status, vaccine type, and duration after vaccination or infection.

## 2. Materials and Methods

### 2.1. LFA Kit and Human Blood Sampling

**Figure 1:**
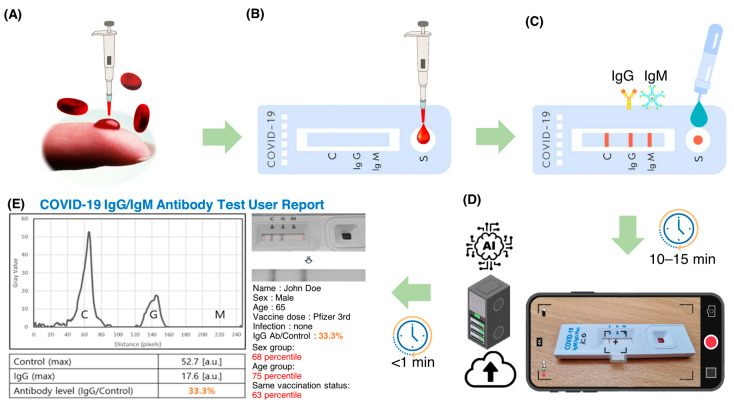
A flowchart illustrating the procedure for AI-based quantitative evaluation of LFA kits. Blood samples were collected from each subject by a single prick of the finger. (**A**) The collected blood samples were placed directly on the sample pad of the test strips. (**B**) A special buffer solution (3–4 drops) was then poured into the sample pad. (**C**) After 10–15 min, the strips were photographed with a smartphone (**D**) and uploaded to a dedicated server. SCAISY quickly analyzed the transferred image (**E**) and provided the user with a report containing the results and statistical data.

Figure 1 shows a flowchart of the steps involved in using an AI-based system for quantitative evaluation of LFA kits. Blood samples (3–5 μL) were collected from the subjects by pricking the finger and directly entered into the sample pad of a COVID-19 IgM/IgG Plus test kit (SD Biosensor, Suwon, Republic of Korea). Whole blood samples were collected with approval from the Institutional Review Board (#2021AN0040) of the Anam Hospital of Korea University. A buffer solution (3–4 drops) provided by the kit manufacturer was added to the sample pad. The sample was then passed through a conjugation pad where antibody and conjugated markers were stored. When the target molecule was present in the sample, it bound to the immobilized conjugated antibodies and markers and continued to move through the LFA kit. As the sample moved through the LFA, the reagents on the nitrocellulose membrane bound to the target molecule on the test line. The presence of the target analyte in the sample was indicated by a colored line formed by light scattering from the GNP, the density of which varied depending on the amount of target analyte present.

### 2.2. Display of Results

The LFA strip used in this study has three detection lines: a control line (C) and two test lines (M and G) for detecting IgM and IgG antibodies, respectively. The control line serves as a safety measure to indicate that the test was performed correctly. A positive result for IgM or IgG antibodies is shown by the appearance of a bright red line on the corresponding M or G test line. The intensity of the color on these test lines reflects the concentration of the analyte in the sample. In case of a negative sample, only the control line (C) shows a red line. 

### 2.3. Data Acquisition Using a Smartphone Camera and Image Analysis

**Figure 2:**
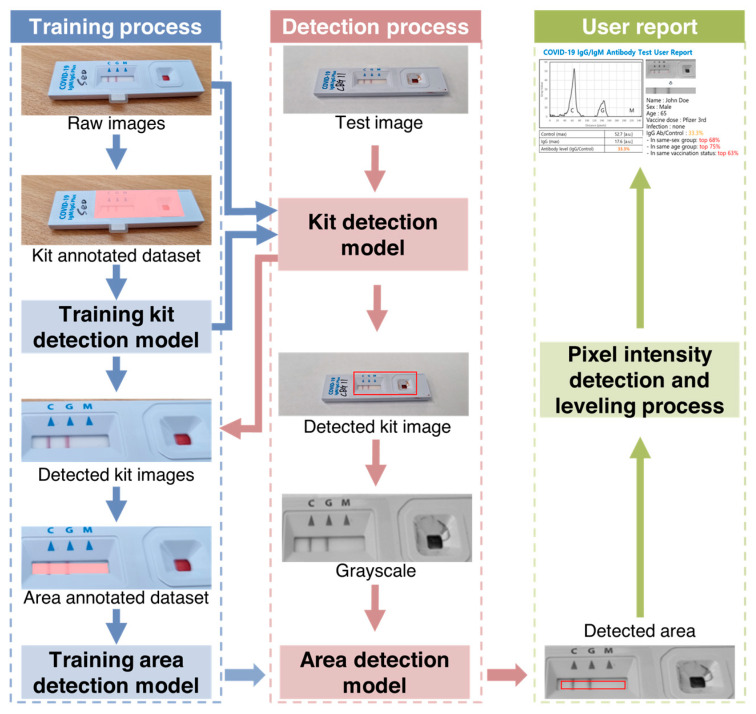
Schematic representation of rapid immunodiagnostic kit image processing for quantitative evaluation of antibody levels using SCAISY. The kit detection algorithm consisted of two mask region-based convolutional neural network (mask-R-CNN) models and a pixel-based quantification function. The two mask-R-CNN models were used to detect the exact area of the kit in the image. The quantification function then extracted and quantified the pixel intensity from the image of the detected area.

We used a smartphone to capture images of the LFA strips 10–15 min after sample application. Images were captured using the rear-facing camera of a Samsung Galaxy S21 smartphone (Samsung Electronics, Seoul, Republic of Korea) running on Android 12.0.0, with autofocus and flash turned off. The images were then uploaded to a server for analysis by an artificial intelligence algorithm. The algorithm quickly analyzed the transferred image and generated a user report with the results and statistical information. Figure 2 shows the workflow of SCAISY.

### 2.4. Feature Extraction

The specialized server for LFA kit evaluation consisted of two main components: a web server that received the kit image and user data after the LFA test, and an analysis server responsible for kit evaluation (Figure S1). During the training process, a range detection model was used to decide the range of the analysis result window. Then, a kit detection model was used to determine whether the image was from a particular LFA kit based on images from different kits. A region of interest containing both test and control lines was automatically extracted from the image. Black and white images were created from the color images and an area detection model was used to extract pixel values from the resulting window image. The user was then presented with a graphical representation of the pixel intensities.

### 2.5. Test to Control Line Signal Intensity (T/C) Quantification Using AI

Figure 2 shows a schematic representation of image processing of rapid immunodiagnostic kits for quantitative evaluation using SCAISY. Two object recognition models based on the mask region-based convolutional neural network (mask-R-CNN) algorithm were used to quantitatively analyze images of kits taken with a camera. The first model recognized the area of the kit in the photo and was trained using images in which the kit area was masked as a polygon. Approximately 700 photos were taken under different environmental conditions to account for factors such as angle and background.

The second model recognized the area used for quantitative analysis within the detected kit area. To improve accuracy, a reference angle was defined for the training dataset and the accuracy of the recognition model was checked when the image was rotated. The area with the highest accuracy among the rotated areas was selected as the final result and converted to a numerical value using a quantification function. A leveling algorithm was used to calibrate this value and correct deviations caused by shadows on the photo.

### 2.6. Comparative Analysis with ELISA

The accurate quantification of SARS-CoV-2-specific IgG antibodies plays a crucial role in assessing immune response and vaccine efficacy. We performed a comparative analysis between SCAISY and ELISA to evaluate their concordance and reliability for quantifying SARS-CoV-2-specific IgG antibody levels. Nineteen blood samples were collected and analyzed using both SCAISY and ELISA methods. In the present study, R- FIND COVID-19 ELISA (SG Medical, Republic of Korea) was used to quantify COVID-19 IgG total antibodies in serum samples, following the manufacturer’s instructions. Serum samples were subjected to a 1:101 dilution using the sample diluent provided in the kit. A volume of 100 μL of the diluted sample was added to a microwell plate coated with the SARS-CoV-2 nucleocapsid protein as antigen. The mixture was then incubated for 45 min at room temperature. After washing the wells with 1× wash buffer, 100 μL IgG conjugate was added to each well and incubated for 30 min at room temperature. After another wash step, 100 µL of TME substrate was added to each well, followed by an incubation period of 15 min in the dark. After the incubation period, a 50 μL stop reagent solution was added to each well. The absorbance of the samples was then measured at 450 nm using an absorbance microplate reader, SunriseTM (Tecan, Zurich, Switzerland). The validity of the study was ensured by using positive and negative controls. The OD450 values from ELISA were compared with the antibody levels calculated from SCAISY. Spearman and Pearson correlation coefficients were calculated to assess the relationship between the ELISA and SCAISY results. Linear regression analysis was performed to determine the extent of the linear relationship. Data were analyzed using GraphPad Prism 9.0.0. (San Diego, CA, USA). 

## 3. Results

### 3.1. Effect of Blood Volume and Measurement Time on Antibody Level

**Figure 3:**
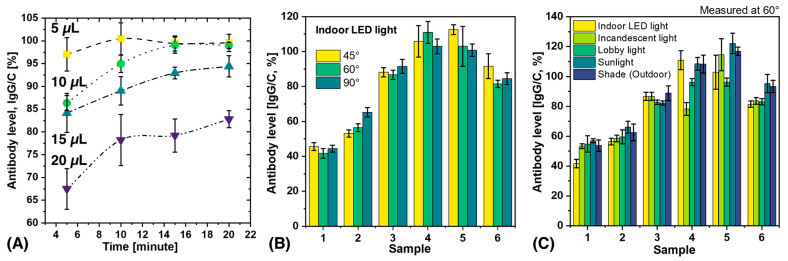
The influence of different factors on the antibody level calculated by SCAISY. (**A**) The influence of blood volume and measurement time on the calculated antibody levels. (**B**) The effect of the acquisition angle on the calculated antibody levels. (**C**) The effect of illumination light on the calculated antibody levels.

Determining the effects of blood volume and measurement time on antibody results is critical for practical applications and also important for scientific replication. In this study, blood volumes of 5–20 µL were applied to the inlet of the test strip, and measurements were performed at intervals of 5–20 min, as shown in Figure 3A. The results showed that antibody levels increased with time and reached a plateau after 10 min. Notably, the results of 5 µL and 10 µL blood were almost identical after 15 min, while the antibody levels decreased when the blood volume was increased to 20 µL. This decrease in antibody levels at a blood volume of 20 µL could be due to the fact that the chemical components of the LFA kit flow through the porous materials and do not reach a steady state. The formation of immune complexes in the test and control lines depends on the finite time for the components to be sufficiently close to each other to combine. This time is determined by the capillary flow rate. Xia et al. have previously reported that sample volume can affect flow rate and that there is an inverse relationship between the effective concentration of analyte in the sample and the square of the change in flow rate [46,47]. Consequently, a larger volume may result in a lower effective antibody concentration as the flow rate of the analyte increases [47]. Therefore, 5–10 µL is the recommended volume and 15 min is the optimal time to obtain reproducible results.

### 3.2. Analysis of Variability Caused by Different Lighting Conditions and Shooting Angles

POCT devices are highly portable and automated diagnostic instruments that can be used in various settings such as hospitals, private homes, outpatient clinics, and remote locations [48,49,50]. These devices can be used in real-world scenarios such as outdoor or indoor camps and by individuals or groups in different environments. For practical applications, it is important to understand the variations that can occur due to different external lighting conditions and image acquisition angles. To evaluate the performance of SCAISY in real-world situations, six different LFA samples were used, and data were collected under five different lighting conditions (indoor LED light, incandescent light, lobby light, sunlight, and shade) and three different camera positions (45°, 60°, and 90°). The influence of the acquisition angle on the results under indoor LED light is shown in Figure 3B, while the influence of the acquisition angle on the results under different lighting conditions is shown in Figure S2. The results for all three angles were almost identical, with a standard deviation (SD) of less than 10%. Thus, SCAISY is a robust system that provides accurate results with a low SD for images captured between 45° and 90°, making it possible to capture images in real-world scenarios without worrying about the camera’s field of view or capture angle. 

The effect of different illumination conditions on the performance of SCAISY was evaluated at two different angles. Figure 3C shows the results obtained at 60° illumination, while Figure S3 shows a comparison of the results under different illumination conditions at 90°. The data show that all illumination conditions gave comparable results within the SD. However, in some cases, antibody levels were slightly lower under incandescent light. However, this deviation is considered negligible since incandescent bulbs are no longer widely used and can be replaced by LED light on a smartphone to avoid this effect. In addition, the reproducibility of the results was evaluated for seven different samples, as shown in Figure S4. For each sample, N images were acquired to evaluate the reproducibility, and the obtained results were transmitted to SCAISY for analysis. The SD was less than 10% under the same illumination conditions, indicating high reproducibility. Furthermore, the repeatability of the results was investigated for four different samples, as shown in Figure S5. The results obtained were consistent, demonstrating the robustness of SCAISY. This feature is particularly useful for medical professionals as it allows them to store digital photos in databases and analyze the results later if needed.

### 3.3. Analysis of Variability Caused by Smartphone Cameras

**Figure 4:**
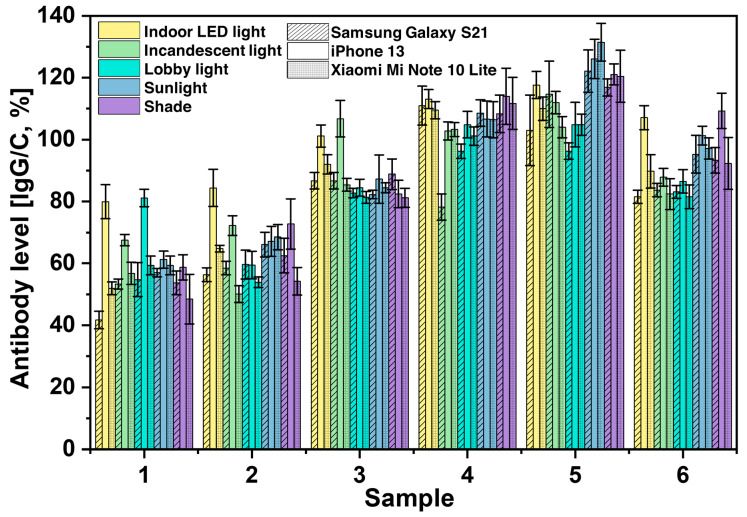
The effects of using different cameras from three different smartphone manufacturers, namely Samsung (represented by stripes), iPhone (represented by no fill pattern), and Xiaomi (represented by a dotted grid), when taking pictures under different lighting conditions. The data show the variations of the calculated antibody values depending on the smartphone brand and lighting conditions.

We also investigated the effects of different cameras on the performance of SCAISY by taking images of six different samples under different lighting conditions (see Figure 4). For the study, we used the rear cameras of two popular Android smartphones, the Samsung Galaxy S21 and the Mi Note 10 Lite (Xiaomi Inc., Beijing, China), and an iPhone 13 (Apple Inc., Cupertino, CA, USA). All phones were equipped with autofocus and the flash was turned off during image capture. The Android phones from Samsung and Xiaomi showed comparable results, with antibody levels within the standard deviation, as shown by the stripes and dotted grid, respectively. In contrast, the iPhone showed marginally higher antibody levels, especially with indoor, incandescent, and lobby lighting for samples 1–3 and with indoor and shade lighting for sample 6. Samsung, on the other hand, showed lower antibody levels under indoor and incandescent lighting for samples 1 and 4, respectively. Despite the slight differences between the phones, it is noteworthy that all phones produced similar antibody levels for the different samples tested. The approximate antibody levels for samples 1–6 were 58%, 60%, 85%, 110%, 120%, and 90%, respectively, and were the same for all light conditions.

The above results suggest that there may be some differences in the performance of different types of cell phones in detecting antibody level with the SCAISY. The difference in results may be due to differences in the camera and image processing capabilities of the various phone models. The sensitivity of the camera to different lighting conditions may also have played a role. It should be noted that our system is designed for POCT where rapid results are important and may not require the same level of accuracy as laboratory-based assays. Furthermore, these potential sources of error can be mitigated by incorporating this information into the AI system and using machine learning to train the AI to recognize and account for these factors, and will be one of the focuses of our future study.

It is worth noting that SCAISY has the potential to be a versatile tool for quantifying several types of LFA kits beyond the scope of this study. By generating a calibration curve via regression analysis using the T/C ratio, SCAISY can estimate the analyte concentration in a sample by analyzing an LFA kit image. Further studies are needed to explore the effectiveness of this approach with different LFA kit types and analytes. Nevertheless, our results are promising evidence for the usefulness of SCAISY as a general approach for quantification of LFA kits and open new possibilities for the application of this technology in clinical and research settings. 

### 3.4. Comparative Analysis of SCAISY and ELISA for Quantification of SARS-CoV-2 Antibody Levels

**Figure 5:**
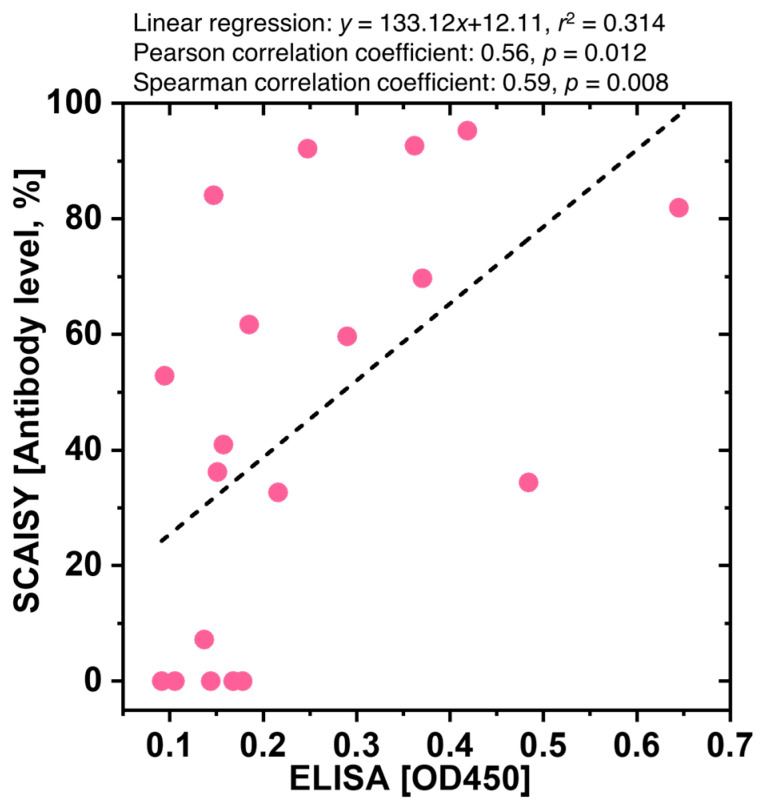
Correlation and linear regression analysis of ELISA and SCAISY results. Scatter plot of optical density values at 450 nm (OD450) for negative and positive samples obtained by the NP -based ELISA protocol plotted against the corresponding antibody levels obtained by SCAISY from LFA images. The *x*-axis represents the OD450 values, while the *y*-axis represents the antibody levels.

The correlations between the OD450 determined by ELISA and the antibody levels determined by SCAISY were tested by calculating Pearson and Spearman correlation coefficients. These results were visualized by a scatter plot for which linear regressions and their slopes and intercepts were calculated (see Figure 5). The *x*-axis represents the optical density for ELISA and the *y*-axis antibody level obtained using SCAISY. ELISA and SCAISY showed a good correlation. Statistical analysis found a correlation between the corresponding values obtained from these tests with *r*^2^ = 0.314 and *p* value = 0.012. The results of the comparative analysis indicate a moderate positive correlation between OD450 levels determined by ELISA and antibody levels calculated by SCAISY. The Spearman correlation coefficient (R_S_) yielded a value of 0.59, whereas the Pearson correlation coefficient (R_P_) yielded a value of 0.56. Statistical significance of both correlations was found with *p* values of 0.012 and 0.008, respectively. In addition, it was observed that certain blood samples showed positive IgG lines on the LFA kit and elevated antibody levels using SCAISY, although low OD450 values were registered by ELISA (Figure S6). The observed incongruence may be attributed to the different capacity of the LFA kit and ELISA assay used in this study. Specifically, the LFA kit can identify antibodies directed against both nucleocapsid protein (NP) and spike protein, whereas the ELISA assay exclusively detects antibodies specific to NP. To compensate for this inconsistency, correlation analysis was performed after excluding samples that showed positive IgG lines despite low OD450 values. The Spearman correlation coefficient increased to approximately 0.83, indicating an increased positive correlation (Figure S7A). The Pearson correlation coefficient increased to approximately 0.81, indicating a positive linear correlation. The results of the adjusted linear regression analysis yielded an improved R-squared value of 0.652, indicating that approximately 65.2% of the variability in antibody levels as determined by SCAISY can be explained by the OD450 values obtained by ELISA after sample removal.

In addition, the correlation between the results was obtained from photographs taken with different cell phone models, namely the iPhone XS and Samsung Galaxy S23 Ultra. The results showed a significant Pearson correlation coefficient of 0.977 and an R-squared value of 0.954, indicating a robust positive linear relationship (Figure S7B). The high correlation observed between the results obtained from images taken by different phone models (iPhone and Galaxy) further emphasizes the consistency and reproducibility of SCAISY.

The results highlight the correlation and agreement between SCAISY and ELISA, considering the different protein targets and the influence of specific samples. The high level of correlation observed between images acquired with different phone models provides confidence in the reliability and consistency of the results. It is essential to conduct further studies and verify these results with a larger population. In addition, exploring alternative variables that may contribute to the observed differences will be an important focus of our future research.

### 3.5. Capabilities of SCAISY

**Figure 6:**
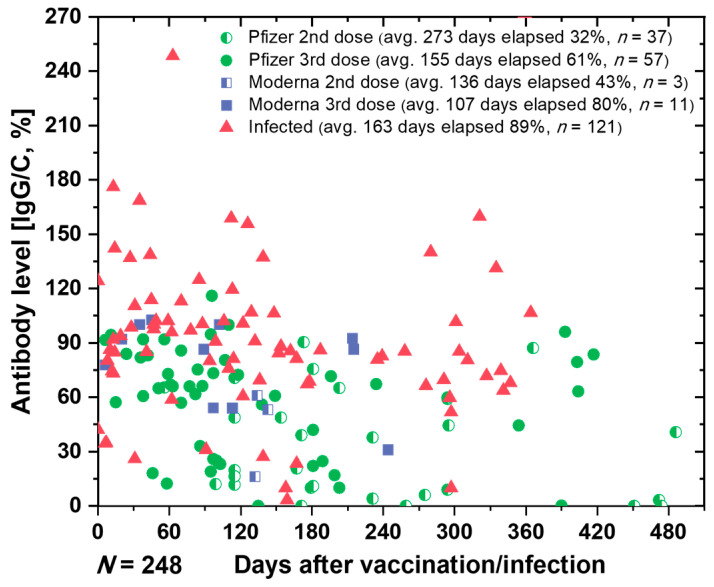
The estimated antibody levels in various individuals.

The primary objective of this study was to perform quantitative measurements of IgG and IgM antibodies, with a focus on developing a rapid, efficient, and cost-effective system for semi-quantitative detection of IgG antibodies. After training SCAISY, we used it to determine the antibody status of more than 240 individuals. Figure 6 shows the SARS-CoV-2 antibody retention rate (IgG/C) over time for various users based on vaccine type and sequence. Our data show that the specific antibody retention rate (IgG/C) for the third dose of Pfizer was 61%, which was 19% lower than that for the third dose of Moderna (80%). In addition, individuals who received a second dose of Pfizer or Moderna (average 273 and 155 elapsed days, respectively) had lower antibody retention rates than individuals who received a third vaccination (average 100–107 elapsed days). 

For individuals who received a third dose of Pfizer vaccine, we grouped the data by 30-day intervals, starting at 0 to 210 days. Figure S8 shows a comparison of antibody levels in different groups of individuals after receiving the third dose of Pfizer, grouped by 30-day intervals. Our data show that relative protection against infection decreased from 81.8% one month after vaccination to 63% three months after the third dose of Pfizer BNT162b2 mRNA COVID-19 vaccine [51]. We observed a gradual decline in antibody levels nearly six months post-vaccination, suggesting that vaccine efficacy declines over time and showing similar trends to previous studies [51].

We want to clarify that our goal is not to draw conclusions about the efficacy of a particular vaccine, but to demonstrate that our method can be used to quantify antibody levels and track them over time. Controlled, randomized trials are needed to reach definitive conclusions about vaccine efficacy and immunity status. By using our method to track an individual’s antibody levels, particularly in the post-COVID-19 era, individuals with low antibody levels may be prioritized for vaccination. This could reduce pressure on the supply chain and ensure an even distribution of vaccinations. In addition, this technology could enable a wider range of individuals to participate in vaccination trials and studies by allowing them to submit their data from the comfort of their own home. 

### 3.6. Monitoring the Antibodies against SARS-CoV-2

**Figure 7:**
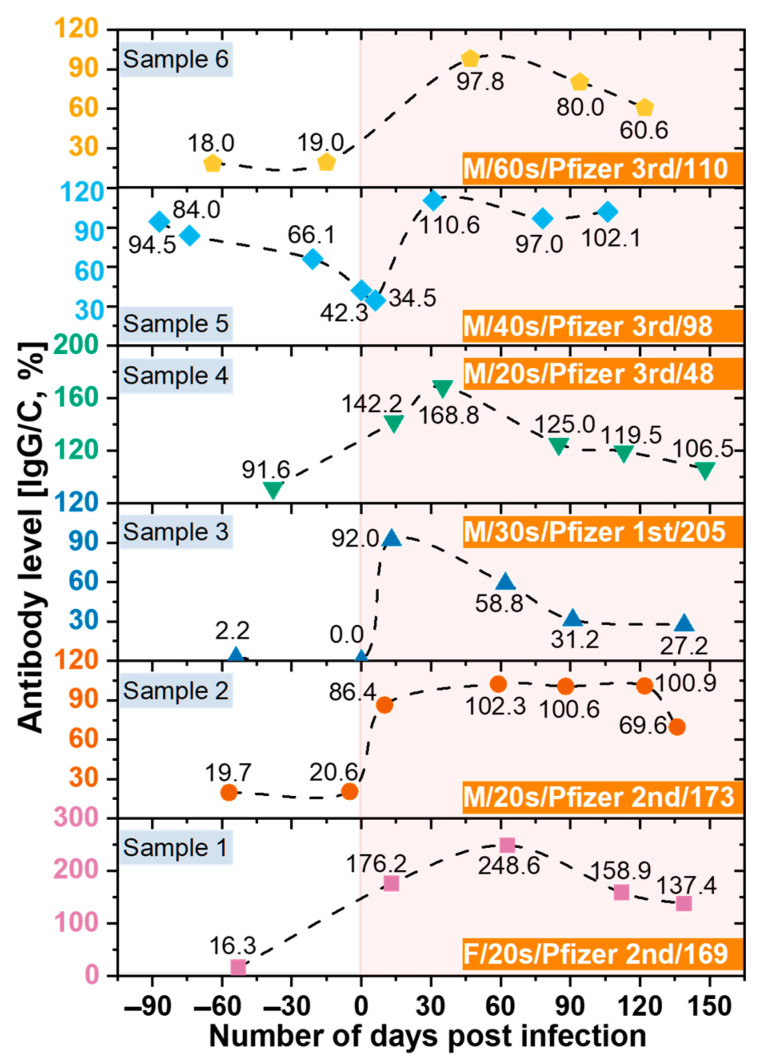
The change in antibody retention rate of confirmed COVID-19 cases over time before and after confirmation of COVID-19. Data were obtained from SCAISY using photographs of an LFA kit taken with a smartphone. Legend indicates sex, age in decades, vaccine manufacturer, and dose, and number of days between vaccination and onset of COVID-19 infection for each individual.

In this study, we also analyzed the changes in antibody retention rate of confirmed COVID-19 cases using SCAISY. As shown in Figure 7, the IgG/C antibody retention rate increased significantly after the COVID-19 confirmation date (day zero) and decreased gradually over time. These results suggest that the immune response to COVID-19 may vary depending on the duration of infection. Interestingly, we observed that antibody levels decreased more slowly in individuals who received the second or third dose of the vaccine, suggesting that vaccination may contribute to the maintenance of long-term immunity against COVID-19. Our results are consistent with previous studies that found a similar decline in antibody response to SARS-CoV-2 over time after infection [52]. However, the potential impact of vaccination on the duration and strength of the immune response to COVID-19 is the subject of ongoing investigation [53,54]. Our study provides further evidence that vaccination may contribute to the maintenance of long-term immunity against COVID-19.

In addition, individuals who received only the first dose of the vaccine had lower peak antibody levels after infection than those who received the second or third dose. The highest antibody level was observed within 15–60 days after infection and gradually decreased after 60 days [53]. These results are consistent with previous studies, including those of Swartz et al., who found that the expected antibody response after COVID-19 infection persisted for more than 500 days after natural infection [54,55]. COVID-19 deaths have been strongly correlated with age. People aged ≥80 years have a 20-fold higher risk of death than people aged <50 years [56]. Furthermore, we found no significant differences in antibody levels between age groups, suggesting that antibody production does not contribute to age-related mortality. However, we acknowledge that underlying health conditions, genetics, and lifestyle factors may also be important for antibody production and function and should be considered when interpreting study results. 

In addition, our study used LFA kits to measure antibody levels, which cannot distinguish between the effects of immunization and the effects of COVID-19 infection. Nonetheless, our focus was on measuring IgG and IgM levels in COVID-19 patients rather than distinguishing between these two effects. We are aware of this limitation and suggest that future studies address this issue. Interestingly, our results are consistent with those of [57], who used an expensive instrument to measure antibody levels in a bead-based multiplex assay (MILLIPLEX^®^ SARS-CoV-2 Antigen Panel 1 IgG; Merck KGaA, Darmstadt, Germany). However, it is important to note that our study was based on a small sample size and further investigation is required to confirm or refute our results.

## 4. Conclusions

In summary, we have shown a feasibility study for the development of SCAISY, a novel POC method for quantification of SARS-CoV-2 IgG antibodies from LFA using a smartphone camera. Our study provides a basis for quantifying and tracking antibody levels over time, comparing levels between individuals, and monitoring immune responses to SARS-CoV-2 exposure or vaccination. We found that images acquired between 45° and 90° provided accurate results with a small standard deviation and that all illumination conditions provided essentially identical results within the standard deviation. In addition, the iPhone camera results should be interpreted with caution, as they yielded higher antibody levels compared to Android phones. Therefore, we recommend that only Android phones be used to estimate antibody levels at this time. Spearman and Pearson correlation coefficients demonstrate a statistically significant correlation between OD450 values determined by ELISA and antibody levels determined by SCAISY. Although this is a proof-of-concept study, we emphasize that randomized controlled clinical trials are needed to ensure accurate quantification of antibody levels for calibration before routine use. Larger sample sizes and more comprehensive assessments of diagnostic accuracy are needed to further validate and optimize the performance of SCAISY. Once the system is calibrated with absolute counts of antibodies, dedicated antibody counting equipment will no longer be needed. SCAISY offers several advantages over traditional methods for detecting antibodies, such as ELISA. Firstly, the system is simple and easy to use, requiring only a smartphone and an LFA kit. Secondly, the SCAISY system provides quantitative data on antibody production rates, allowing accurate tracking of changes in immune responses over time. Finally, the SCAISY system can be deployed in resource-limited environments, making it a valuable tool for public health and real-time health monitoring. Therefore, SCAISY has tremendous potential for use in clinical and community health settings in developing countries, as well as for direct-to-consumer market. In conclusion, SCAISY is a promising tool for rapid and accurate serodiagnosis of COVID-19 and has the potential to facilitate early detection of infection, guide vaccination interventions, and monitor the effectiveness of interventions.

## Data Availability

The data presented in this study are available on request from the corresponding author.

## Supplementary Materials

The following supporting information can be downloaded at: https://www.mdpi.com/article/10.3390/bios13060623/s1, Figure S1: The system architecture of the platform that users upload their images to the server and receive a quantified result report; Figure S2: The influence of the shooting angle on the result under different lighting conditions; Figure S3: A comparison of the different illumination situations at 90°; Figure S4: The antibody levels and standard deviation of antibody levels for seven different samples; Figure S5: The repeatability of results for four different samples. Reproducibility and repeatability results; Figure S6: Lateral flow assay images and corresponding optical density 450 (OD450) from ELISA and antibody levels values obtained with SCAISY; Figure S7: (A) Correlation and linear regression analysis of ELISA and SCAISY results after excluding samples that showed positive IgG lines despite low OD450 values. (B) The correlation between the results obtained from photographs taken with iPhone XS and Samsung Galaxy S23 Ultra; Figure S8: A comparison of antibody levels in individuals after receiving the third dose of a COVID-19 vaccine in a 30-day group.
