# Supplementary material for: Proof-of-Concept: Smartphone- and Cloud-Based Artificial Intelligence Quantitative Analysis System (SCAISY) for SARS-CoV-2-Specific IgG Antibody Lateral Flow Assays
Source: Biosensors (Basel). 2023 Jun 5;13(6):623. doi: 10.3390/bios13060623 (PMC10296264; doi:10.3390/bios13060623)
Supplement: Supplementary file 1 [file biosensors-13-00623-s001.zip › biosensors-2383366-supplementary.pdf]

biosensors

MDPI

Supplemental Information

Proof-of-Concept: Smartphone- and Cloud-Based Artificial Intelligence Quantitative Analysis System (SCAISY) for SARS-CoV-2-Specific IgG Antibody Lateral Flow Assays

Samir Kumar †, Taewoo Ko †, Yeonghun Chae, Yuyeon Jang, Inha Lee, Ahyeon Lee, Sanghoon Shin, Myung-Hyun Nam, Byung Soo Kim, Hyun Sik Jun * and Sungkyu Seo *

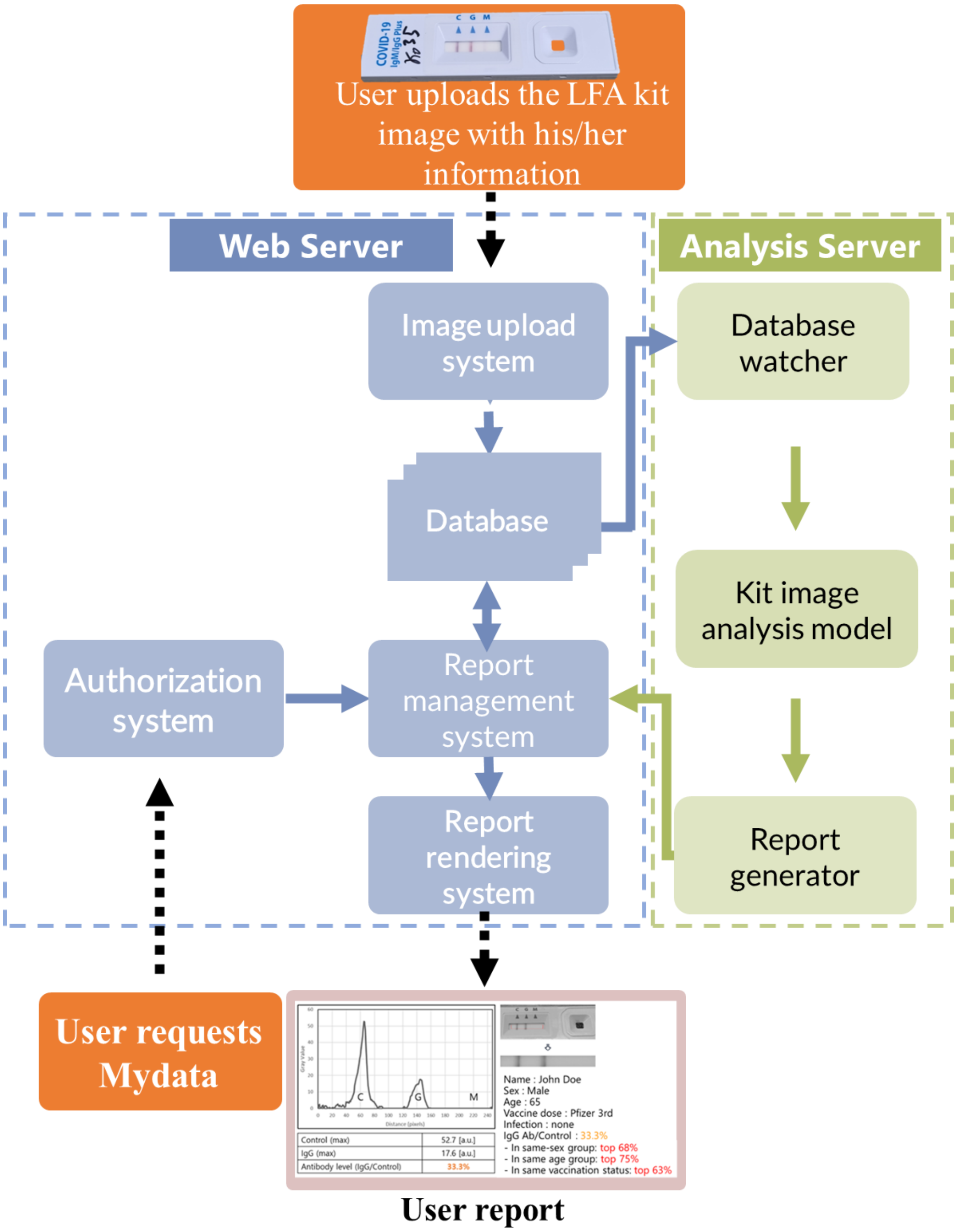

Figure S1. The system architecture of the platform with which users upload their images to the server and receive a quantified result report. User images are sent to the server through the image upload system and then stored in the database. The analysis server watches the user's upload event. When a new image is uploaded, the result is derived through the deep-learning model, and then

sent to the web server. The results transmitted from the analysis server to the web server are stored in the database through the report management system and processed into a form to be displayed to users. By access using authorization system, users can check their earlier results.

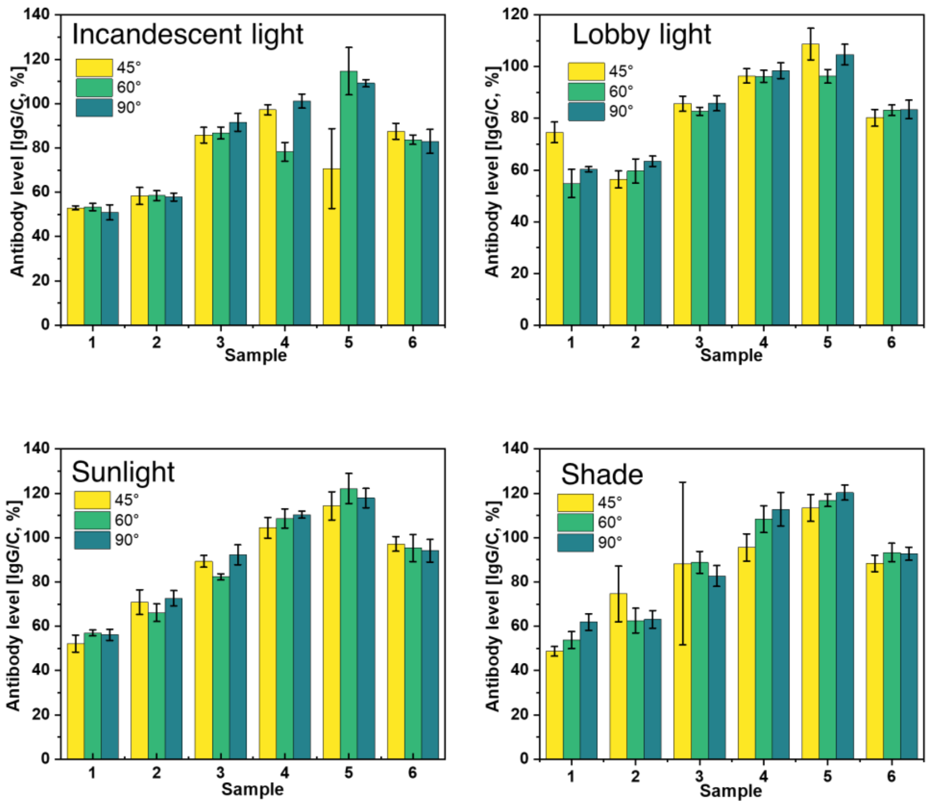

Figure S2. The influence of the shooting angle on the results under different lighting conditions.

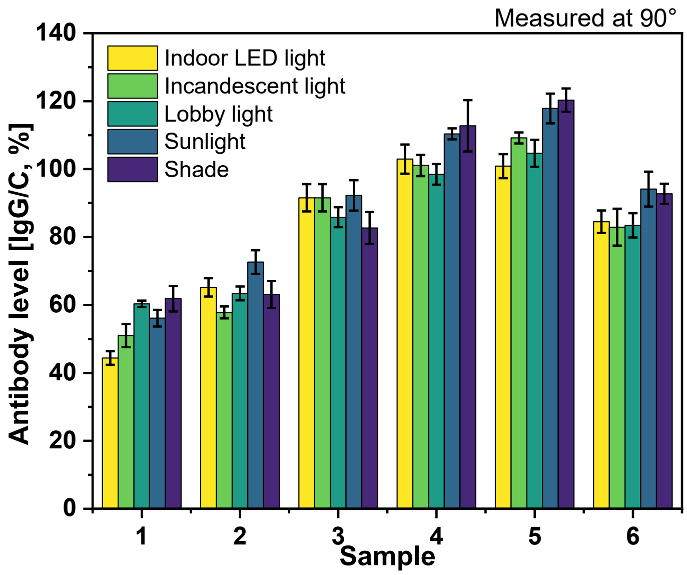

Figure S3. A comparison of the different illumination situations at 90°.

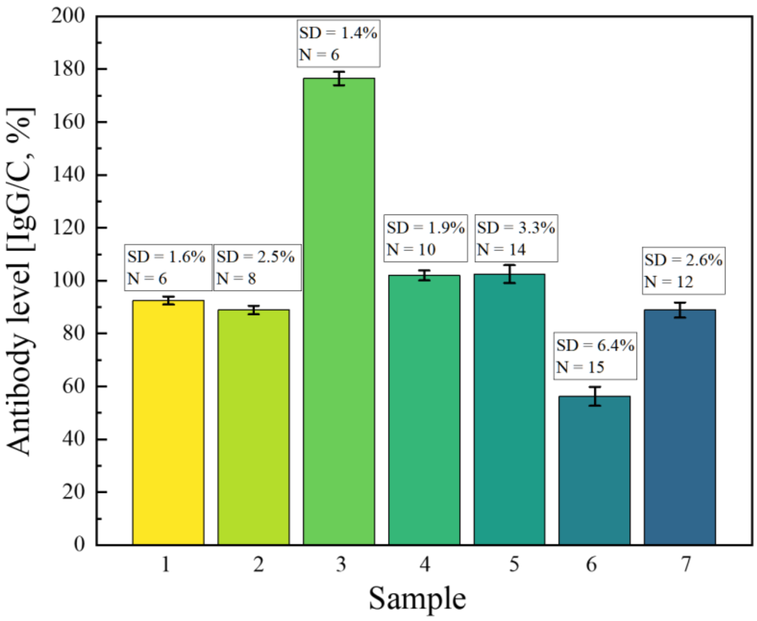

Figure S4. The antibody levels and standard deviation of antibody levels for seven different samples.

Using a different image from the same LFA kit, we assessed the reproducibility of our AI-based LFA measurement to ensure that the results were consistent, regardless of the image used. Figure S4 shows the antibody levels and standard deviations for seven different samples. N images were acquired for seven different samples for the reproducibility test and then transmitted to SCAISY to determine antibody levels. Under the same illumination and settings, the SD was less than 10%.

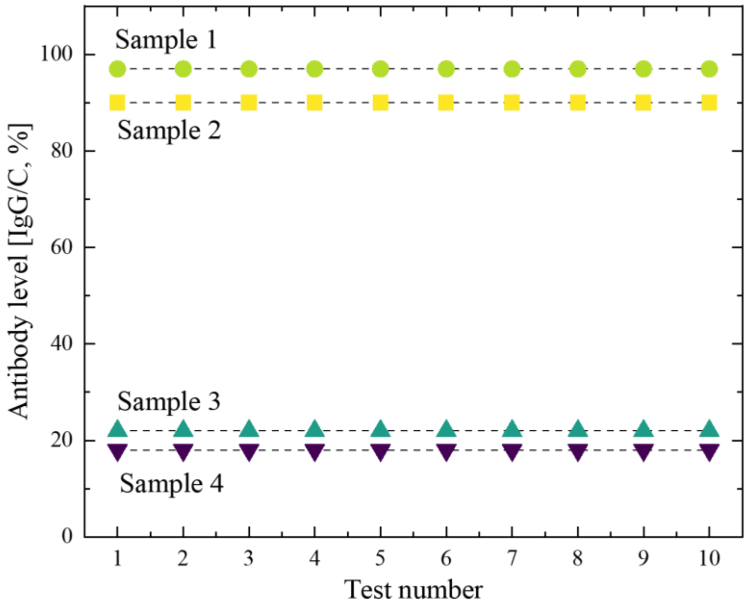

Figure S5. The repeatability of results for four different samples.

Reproducibility and repeatability results

To evaluate the precision of our SCAISY system, a repeatability study was conducted by comparing the absolute differences between the same image uploads at different time points for antibody level determination. Simply put, we needed to decide whether our system always yields the same results when exposed to the same image (experiment).

Figure S5 shows the repeatability of the results for four different samples. As expected, SCAISY provided similar results. This is impressive because it allows healthcare professionals to store digital photographs in databases and analyze the results later if needed.

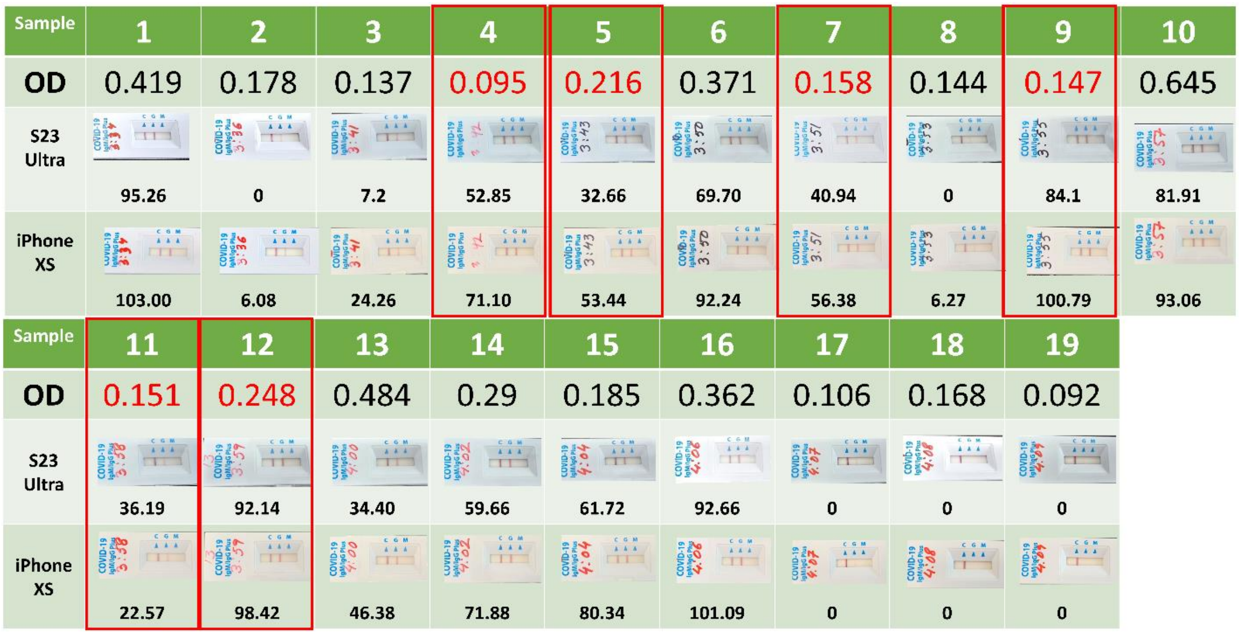

Sample	1	2	3	4	5	6	7	8	9	10
OD	0.419	0.178	0.137	0.095	0.216	0.371	0.158	0.144	0.147	0.645
S23 Ultra	95.26	0	7.2	52.85	32.66	69.70	40.94	0	84.1	81.91
iPhone XS	103.00	6.08	24.26	71.10	53.44	92.24	56.38	6.27	100.79	93.06

Sample	11	12	13	14	15	16	17	18	19
OD	0.151	0.248	0.484	0.29	0.185	0.362	0.106	0.168	0.092
S23 Ultra	36.19	92.14	34.40	59.66	61.72	92.66	0	0	0
iPhone XS	22.57	98.42	46.38	71.88	80.34	101.09	0	0	0

Figure S6. Lateral flow assay images and corresponding optical density 450 (OD450) from ELISA and antibody levels values obtained with SCAISY. Images were acquired using a Galaxy S23 Ultra and an iPhone XS. A few blood samples (within red boxes) showed positive IgG lines on the lateral flow assay (LFA) kit and elevated antibody levels using SCAISY, although low OD450 values were registered by ELISA. The observed incongruence may be attributed to the different capacities of the LFA kit and ELISA assay used in this study. Specifically, the LFA kit can identify antibodies directed against both nucleocapsid protein (NP) and spike protein, whereas the ELISA assay exclusively detects antibodies specific to NP.

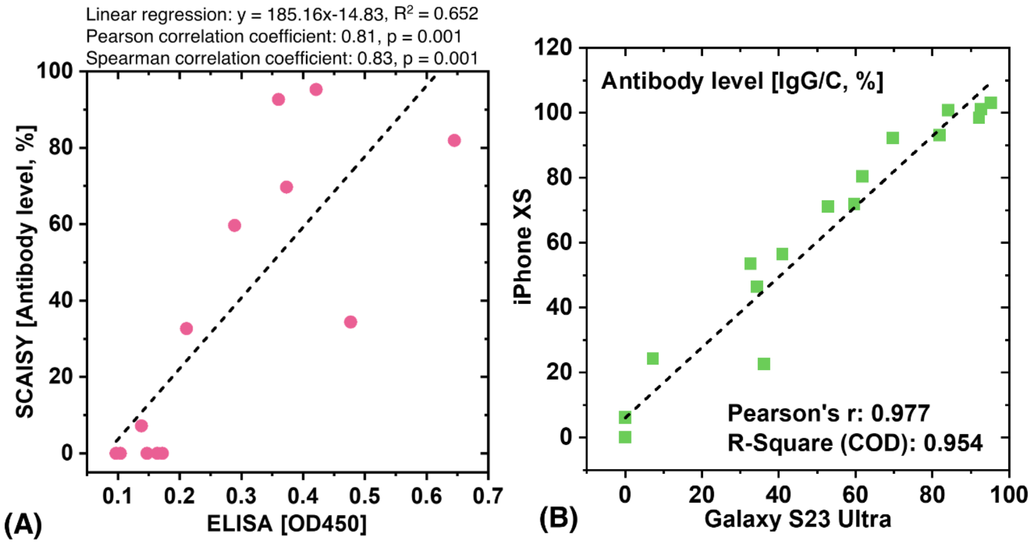

Figure S7. (A) Correlation and linear regression analysis of ELISA and SCAISY results after excluding samples that showed positive IgG lines despite low OD450 values. Scatter plot of optical density values at 450 nm (OD450) for negative and positive samples obtained by the NP-based ELISA protocol plotted against the corresponding antibody levels obtained by SCAISY from LFA images. The *x*-axis is the OD450 values, while the *y*-axis represents the antibody levels. (B) The correlation between the results was obtained from photographs taken with iPhone XS and Samsung Galaxy S23 Ultra.

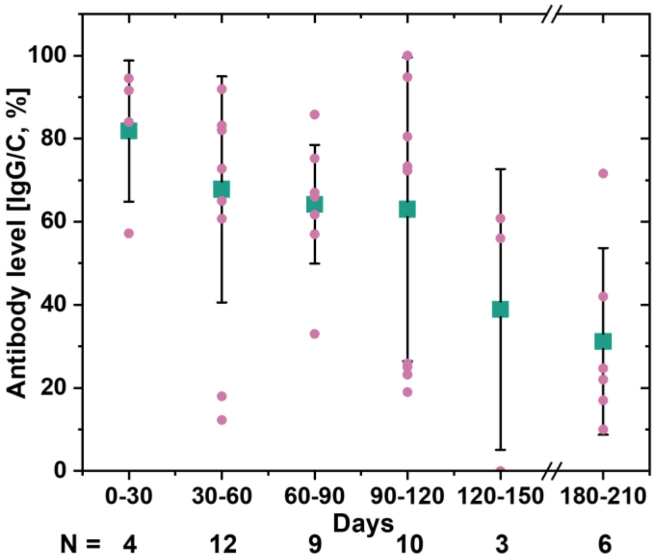

Figure S8. A comparison of antibody levels in individuals after receiving the third dose of a COVID-19 vaccine in a 30-day group.
